# Ethanol Affects the Development of Sensory Hair Cells in Larval Zebrafish (*Danio rerio*)

**DOI:** 10.1371/journal.pone.0083039

**Published:** 2013-12-06

**Authors:** Phillip M. Uribe, James D. Asuncion, Jonathan I. Matsui

**Affiliations:** 1 Department of Neuroscience, Pomona College, Claremont, California, United States of America; 2 Department of Molecular and Cellular Biology, Harvard University, Cambridge, Massachusetts, United States of America; 3 Department of Otolaryngology and Communication Enhancement, Boston Children’s Hospital, Boston, Massachusetts, United States of America; Biogen Idec, United States of America

## Abstract

Children born to mothers with substantial alcohol consumption during pregnancy can present a number of morphological, cognitive, and sensory abnormalities, including hearing deficits, collectively known as fetal alcohol syndrome (FAS). The goal of this study was to determine if the zebrafish lateral line could be used to study sensory hair cell abnormalities caused by exposure to ethanol during embryogenesis. Some lateral line sensory hair cells are present at 2 days post-fertilization (dpf) and are functional by 5 dpf. Zebrafish embryos were raised in fish water supplemented with varying concentrations of ethanol (0.75%–1.75% by volume) from 2 dpf through 5 dpf. Ethanol treatment during development resulted in many physical abnormalities characteristic of FAS in humans. Also, the number of sensory hair cells decreased as the concentration of ethanol increased in a dose-dependent manner. The dye FM 1-43FX was used to detect the presence of functional mechanotransduction channels. The percentage of FM 1-43-labeled hair cells decreased as the concentration of ethanol increased. Methanol treatment did not affect the development of hair cells. The cell cycle markers proliferating cell nuclear antigen (PCNA) and bromodeoxyuridine (BrdU) demonstrated that ethanol reduced the number of sensory hair cells, as a consequence of decreased cellular proliferation. There was also a significant increase in the rate of apoptosis, as determined by TUNEL-labeling, in neuromasts following ethanol treatment during larval development. Therefore, zebrafish are a useful animal model to study the effects of hair cell developmental disorders associated with FAS.

## Introduction

Consumption of alcoholic beverages during pregnancy increases the risk of fetal alcohol syndrome (FAS) - a collection of physical and cognitive problems including craniofacial defects, visual and auditory deficits, impaired motor skills, and learning deficits [1]. Although many studies have shown the adverse effects of alcohol on the developing fetus, FAS is still prevalent today at a rate of ∼2 per 1000 live births in the United States [2], [3]. Auditory dysfunction occurs in approximately 30% of the children diagnosed with FAS [2], [3]. Interestingly, clinical studies have reported no conclusive evidence of vestibular dysfunction despite the commonalities between the auditory and vestibular systems [4]. Nevertheless, human studies to investigate FAS are problematic due to a wide variability in dose, exposure, duration, and response measures [5]. Moreover, there are many other confounding variables, including misreporting of alcohol consumption or additional activities that are detrimental to the developing fetus.

Due to this inherent variability, animal models provide scientific rigor for investigations of prenatal alcohol exposure. In murine models of FAS, ethanol induces malformed developing sensory hair cells with ultrastructural defects including disintegrating cytoplasm and organelles, rendering the sensory cells nonfunctional [6]. Moreover, embryonic rats treated with ethanol have dysfunctional auditory brain stem responses that are associated with missing sensory hair cells and malformed stereocilia bundles [7]–[10] while embryonic mice treated with ethanol have excessive cell death and fail to develop inner ears [11]. Conflicting observations of dysfunction in the vestibular system in rodent models following prenatal ethanol exposure offer no consistent conclusions [4]. Although experimental data have been obtained from using mouse and rat studies, there are many difficulties in administering and maintaining alcohol levels in mammals [5].

One of the challenges of analyzing ethanol’s teratogenicity in rodent model systems is that embryos develop *in utero*. Therefore, ethanol concentrations and exposure times are very difficult to determine, since the metabolic status of the mother needs to be also considered. Other vertebrates, such as *Xenopus laevis* and zebrafish (*Danio rerio*) develop *ex utero*, allowing specific concentrations of ethanol over specific developmental periods to be administered. Treating *Xenopus* and zebrafish embryos with ethanol results in phenotypes comparable to those described for FAS children, suggesting that the same molecular mechanisms occur in mammals, amphibians, and fish [12]–[16]. Although multiple studies have demonstrated that alcohol affects the development of the zebrafish visual system [1], [13], [15]–[19], little is known about how ethanol affects the development of zebrafish sensory hair cells [20]. In one study, embryonic zebrafish treated with ethanol resulted in abnormal otolith development, decreased saccular hair cell density, and fewer hair cells per neuromast [20]. Nevertheless, the mechanism regulating the decreased number of hair cells has yet to be determined.

The goal of this study was to test the effect of ethanol on neuromast cellular proliferation and sensory hair cells differentiation in zebrafish neuromasts. We demonstrate that zebrafish embryos treated with ethanol have morphological abnormalities described in FAS rodent models. Furthermore, we show that ethanol specifically inhibits the production of sensory hair cells and that this inhibition correlates with a reduction in hair cell function along with an increase in the number of apoptotic cells.

## Materials and Methods

### Breeding fish and treating zebrafish larvae with ethanol

We used either *AB wildtype strain (Zebrafish International Resource Center, Eugene, OR) or TG(Brn3c:GAP43-GFP)^s356t^ transgenic fish (Dr. Herwig Bayer, Max-Plank Institute for Neurobiology, Munich, Germany) that endogenously express green fluorescent protein (GFP) in the plasma membrane of sensory hair cells under the control of the *pou4f3 (brn3c*) promoter [21]–[23]. These adult zebrafish were bred and maintained using standard procedures as inbred stocks on a 14 hour light/10 hour dark cycle in the Harvard University and the Pomona College zebrafish facilities [24]. Embryos were manually staged using standard morphological criteria [25]. The embryos were raised in embryo medium (EM) until 48 hours post-fertilization (hpf) in a 28.5°C incubator and then transferred to a 6-well plate containing EM and varying concentrations of USP grade ethanol (0.75%–1.75% by volume, Pharmco Products, Brookfield, CT) or 1%–1.5% methanol by volume (Sigma-Aldrich Corporation, St. Louis, MO). The EM, ethanol, and methanol were changed on a daily basis until 5 dpf, when larvae were fixed with 4% paraformaldehyde at room temperature and then rinsed with phosphate-buffered saline (PBS; Sigma-Aldrich) 3 times for 5 minutes each. In order to assess whether hair cells could develop after ethanol treatment ended, some larvae were rinsed several times with EM, raised in EM until 10 dpf, and then fixed in paraformaldehyde. The EM were changed daily and larvae were fed Hatchfry Ecapsulon (Grade 0; 30 μm; Argent Laboratories, Redmond, WA).

The Pomona College Institutional Animal Care and Use Committee (IACUC; Animal assurance number A3605-01) and the Boston Children’s Hospital IACUC (Animal assurance number A3303-01) approved all of the experimental protocols, which conform to the National Institutes of Health animal use guidelines.

### Fluorophores and antibodies

All staining procedures were performed at room temperature unless otherwise noted. The following reagents were obtained from Life Technologies (Carlsbad, CA): 0.15% FM 1-43FX in EM; Alexa Fluor 488 phalloidin (1:100 in PBS); Click-iT TUNEL Alexa Fluor 594 imaging assay kit. The following antibodies were used and all were prepared in blocking solution (see below). Rabbit anti-Myosin-VI primary antibody (1∶500; Proteus Biosciences, Ramona, CA); mouse anti-PCNA primary antibody (1∶500; Sigma-Aldrich); mouse anti-BrdU primary antibody (1∶100; BD Biosciences, San Jose, CA); Alexa Fluor 488 goat-anti-rabbit IgG secondary antibody (1∶500; Life Technologies); Alexa Fluor 594 conjugated goat-anti-mouse IgG secondary antibody (1∶200; Life Technologies).

### FM 1-43 staining to identify functioning lateral line hair cells and phalloidin staining

The fluorescent dye FM 1-43FX was used to label hair cells with functioning mechanotransduction channels within the lateral line neuromasts [26], [27]. At 5 dpf, larvae were anesthetized with MS-222 (Sigma-Aldrich) [24] and incubated in EM containing 0.15% FM 1-43FX for 30 seconds. Larvae were then rinsed with EM three times for 30 seconds each and then fixed in 4% paraformaldehyde and rinsed with PBS, mounted, and imaged. The percentage of FM 1-43 labeled cells that were co-labeled with GFP was calculated.

### Myosin VI immunohistochemistry

When wild-type zebrafish were used for experiments, tissue was immunolabeled for Myosin VI to detect sensory hair cells [28]. Embryos were permeabilized with PBST 3 times for 20 minutes each. Next, they were rinsed with distilled water for 30 minutes. Embryos were incubated in blocking solution (1% bovine serum albumin, 5% normal goat serum, 1% DMSO in PBST) for 1 hour and then immediately incubated in rabbit anti-Myosin-VI antibody overnight at 4°C. The tissue was then washed with PBST 3 times for 10 minutes each. Embryos were then incubated in blocking solution for 1 hour and then incubated with secondary antibody for 4 hours in the dark. Embryos were rinsed with PBS 3 times for 5 minutes each. Some larvae were co-labeled for BrdU detection (see below). Prior to additional immunohistochemistry, specimens were fixed in 4% paraformaldehyde for 20 minutes and rinsed with PBS 3 times for 5 minutes each.

### Proliferating cell nuclear antigen (PCNA) immunohistochemistry

To measure relative levels of cell proliferation, larvae were fixed at 3, 4, and 5 dpf and immunolabeled for PCNA [29]. Larvae were permeabilized with methanol for 1 hour at –20°C then rinsed with PBS. Tissue was then permeabilized again with acetone for 5 minutes, rinsed with distilled water, and rinsed with 0.1% Tween-20 plus PBS twice for 5 minutes each. Specimens were then incubated in blocking solution (20% lamb serum, 1% DMSO, 0.1% Tween-20 in PBS) for 1 hour. Next, specimens were incubated with mouse anti-PCNA antibody overnight at 4°C. The antibody was washed away the next day with PBS plus 0.1% Tween-20 3 times for 10 minutes each. The tissue was then incubated in blocking solution for 30 minutes. Next, larvae were incubated in secondary antibody for 4 hours in the dark and then rinsed 3 times with PBS for 5 minutes each.

### Bromodeoxyuridine (BrdU) treatment and immunohistochemistry

Cell proliferation was also measured by uptake and detection of BrdU [28]. Embryos were incubated in embryo media containing 10 mM BrdU (Sigma) and 1% DMSO for 24 hours prior to fixation and then fixed in paraformaldehyde as described above. Tissue was permeabilized with PBDT (PBS, 1% DMSO, 0.1% Tween-20) three times for 10 minutes each and then dehydrated in 100% methanol for 1 hour at –20°C. Next, embryos were rehydrated in a graded series of decreasing methanol concentration in PBS. The larvae were then washed with PBDT for 10 minutes. Embryos were then digested with proteinase K (10 ug/ml; Sigma-Aldrich) for 20 minutes and then re-fixed in 4% paraformaldehyde for 20 minutes. Embryos were rinsed 3 times with PBDT for 10 minutes each and then incubated in 1N hydrochloric acid (Sigma-Aldrich) for 1 hour. The embryos were then rinsed with PBDT 3 times for 10 minutes. Next, the tissue was incubated in blocking solution (10% normal goat serum in PBDT) for 1 hour. The embryos were then incubated with mouse anti-BrdU overnight at 4°C and then rinsed with PBDT 3 times for 10 minutes each. The larvae were then incubated with blocking solution for 1 hour followed by the secondary antibody for 4 hours in the dark. Finally, the embryos were rinsed with PBS 3 times for 5 minutes.

### TUNEL labeling

In order to assess relative levels of apoptosis, larvae were fixed at 3, 4, or 5 dpf and processed for TUNEL labeling (Terminal dUTP Nick-End-Labeling) [30]. Briefly, fixed larvae were digested with 10 µg/mL proteinase K for 40 minutes. They were then refixed in 4% paraformaldehyde for 20 minutes and then rinsed with PBS three times for ten minutes each. The larvae were processed using a Click-iT TUNEL Alexa Fluor 594 imaging assay kit following the directions supplied by the manufacturer.

### Imaging

All specimens were whole mounted in either Vectashield (Vector Labs, Burlingame, CA) or glycerol/PBS (9:1). Images of lateral line neuromasts were obtained using a Leica TCS SP confocal laser-scanning microscope (Leica Microsystems, Heidelberg, Germany) or a Nikon-C1-SI confocal microscope (Nikon Instruments Inc., Melville, NY). Single images and compressed *z*-series were collected with Leica Software (Leica Microsystems) or EZ-C1 software (Nikon Instruments). Cell counts were performed at the time of imaging by viewing the image slices sequentially. Alternatively, images were obtained using a Nikon Eclipse Ni Fluorescence Microscope (Nikon Instruments) using a 60X objective and video images were obtained using a Nikon DS-Qi1 Cooled CCD camera and NIS Elements software (Nikon Instruments). Both the O2 and Mi1 neuromasts were imaged for all of the experiments. These neuromasts were studied since they are easily identifiable and have been used in previous studies [23], [31]. The entire neuromast can be observed in one field of view when the larval zebrafish is mounted on its side. Images were scaled and cropped using Adobe Photoshop (Adobe Systems, San Jose, CA), ImageJ (National Institutes of Health, Bethesda, MD), or Nikon EZ-C1 software.

### Statistical analysis

Statistical analyses were performed using either an unpaired two-sample *t*-test assuming unequal variances (Excel; Microsoft Corporation, Redmond, WA), a one-way analysis of variance (ANOVA; Statistica, StatSoft Inc., Tulsa, OK), or VassarStats (Vassar College, Poughkeepsie, NY). Post-hoc comparisons, when appropriate, utilized the Tukey-Kramer HSD test. Alternatively, the data were analyzed using analysis of covariance (ANCOVA) using SPSS (IBM Corporation, Armonk, NY).

## Results

### Ethanol treatment affects the development of sensory hair cells in the lateral line

**Figure 1:**
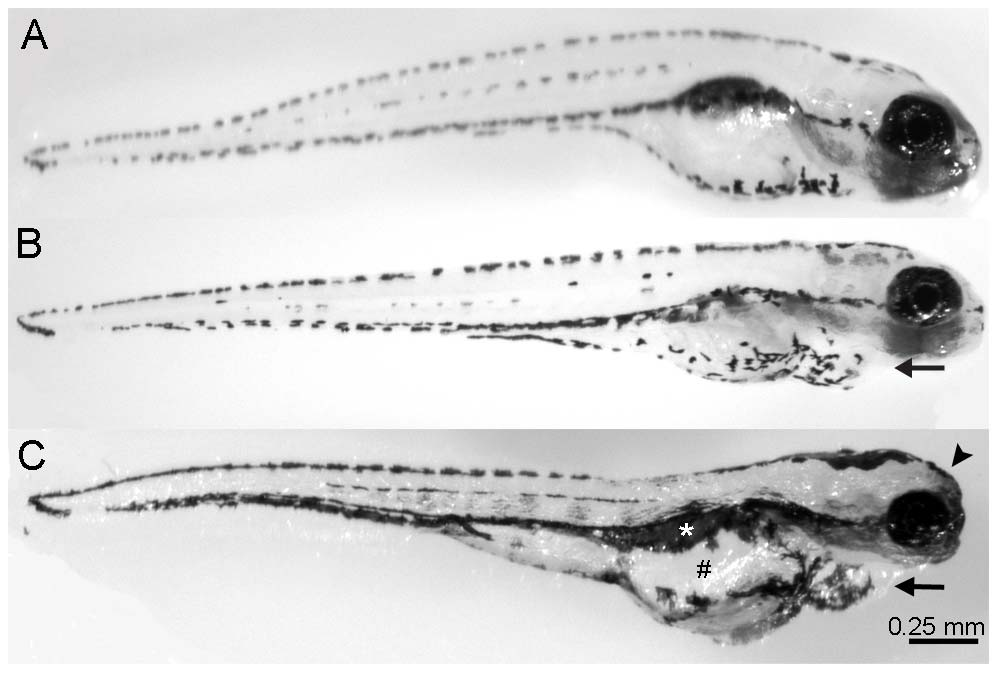
Ethanol treatment affects the development of larval zebrafish. Zebrafish embryos were raised in varying concentrations of ethanol (0% through 1.5% by volume) from 2 days post-fertilization (dpf) to 5 dpf, fixed, and imaged. (A) Untreated control animals appear normal. (B) 1% ethanol-treated animals have a slightly swollen heart (arrow) and no swim bladder but otherwise appear normal. (C) Increasing the concentration of ethanol treatment to 1.5% resulted in embryos exhibiting swollen hearts (arrow), swollen guts (#), missing a swim bladder (white asterisk), and inhibited craniofacial development (arrowhead), which is similar to the phenotype exhibited by children with FAS.

Zebrafish embryos were raised in embryonic medium (EM) supplemented with varying concentrations of ethanol (0.75%–1.75% by volume) from 2 days post-fertilization (dpf) through 5 dpf since we wanted to investigate how ethanol affects the development of lateral line hair cells [31]–[33]. Some hair cells are present at 2 dpf but most of the hair cells are added by 5 dpf and are functional at that time [31]–[33]. Untreated controls (Fig. 1A) and larvae receiving 0.75% ethanol appeared morphologically normal (data not shown). Larvae receiving 1% ethanol had slightly swollen hearts (arrow, Fig 1B) and no swim bladders but otherwise appeared normal (Fig. 1B). Embryos treated with concentrations of 1.25% ethanol and greater began to show different morphological characteristics including a slightly flatter forebrain, a noticeably swollen heart, and swollen gut (data not shown). Embryos treated with 1.5% and 1.75% ethanol had numerous morphological problems including an extremely swollen heart with blood sometimes pooling in the chamber (arrow, Fig. 1C), rounded forebrain (arrowhead), no swim bladder (white asterisk), swollen gut (#), irregular jaw, and smaller eyes. These observations are consistent with previously reported work when ethanol was present during the same developmental time period [16]. In some cases, high concentrations of ethanol resulted in a dorsal curve to the body (data not shown). Embryos treated with higher concentrations of ethanol were often listless and only swam after being touched with a probe. All of the zebrafish had normal touch responses and beating hearts. Larvae treated with the same concentrations of ethanol at 1 dpf through 5 dpf had more severe morphological defects (data not shown).

**Figure 2:**
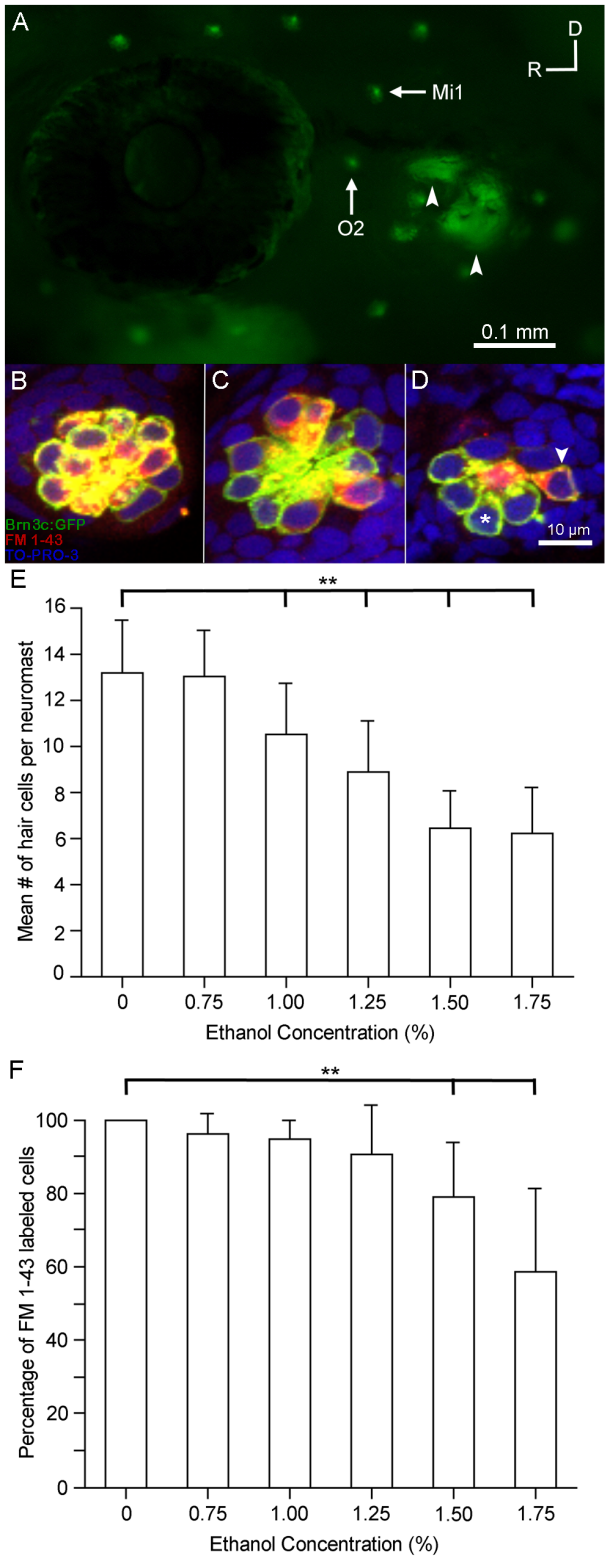
Ethanol but not methanol treatment affects the number of functional sensory hair cells in the lateral line. Brn3c-GFP transgenic zebrafish embryos were raised in embryo medium supplemented with varying concentrations of ethanol. Prior to fixation, the larvae were stained with FM 1-43FX (red) to determine if hair cell mechanotransduction channels were functional. (A) Lateral view of a Brn3c-GFP zebrafish. Clusters of bright green GFP-labeled hair cells are found in neuromasts along the head and body of the animal. The O2 and Mi1 neuromasts are highlighted along with inner ear organs (small arrows). Higher magnification of hair cells of the O2 neuromast taken from *z*-stacks show that (B) hair cells in untreated controls and (C) 1% ethanol-treated animals had no observable morphological differences though there were fewer hair cells in the 1% ethanol-treated animals. Almost all of the GFP-labeled hair cells were co-stained with FM 1-43FX. (D) Fewer sensory hair cells were observed in neuromasts of animals treated with 1.5% ethanol. There were also fewer double-labeled cells (arrowhead) and more single labeled cells (asterisk). (E) Stereocilia bundles were counted to determine the number of hair cells present in the O2 neuromast. Control numbers were similar to those reported in other studies [23], [31]. Significantly fewer hair cells (**p<0.01) were counted in ethanol-treated larvae at concentrations greater than 1% per volume when compared to controls. No differences in the number of hair cells were observed in larvae treated with 1.5% methanol. (F) The percentage of GFP-labeled cells that were also co-stained for FM 1-43 decreased as the concentration of ethanol increased but not in methanol-treated larvae. There was a significant decrease (**p<0.01) in the number of double-labeled hair cells at the two highest concentrations of ethanol tested. Results are the mean values ± SD. n = 8-21 per condition.

We examined the effects of ethanol on the development of sensory hair cells in the neuromasts found in the lateral line. By 5 dpf, the lateral line consists of neuromasts that reside along the head and body in a stereotyped manner (Fig. 2A) [32], [34]. Each neuromast contains a central cluster of hair cells that function to detect water current relative to the animal’s body via movement of their stereocilia [35], [36]. The mean numbers of hair cells contained in each neuromast differ greatly depending on the location in the lateral line system and when they appear in development [31], [32]. We counted the number of hair cells in the otic 2 (O2) neuromasts of untreated Brn3c-GFP zebrafish (Fig. 2A, B, green hair cells) and found that the O2 neuromast had 13.1±2.3 hair cells (n = 21) which are consistent with other published results [23], [31].

The hair cells in zebrafish exposed to 0.75% or 1% ethanol (Fig. 2C) closely resembled those of untreated controls. Each neuromast could be distinguished clearly and the placement of the neuromasts around the eye and throughout the rostral head and dorsal areas were similar to untreated controls. Embryos treated with 1.25% ethanol had missing neuromasts and neuromast cytoarchitecture was more severely impacted in animals treated with 1.5% ethanol (Fig. 2D). There were significantly fewer hair cells in ethanol-treated embryos at concentrations at or greater than 1% in the O2 neuromast (p<0.01; Fig. 2E).

We also examined the middle 1 (Mi1) neuromast. In untreated control animals, the Mi1 neuromast had 14.2±3.8 hair cells (n = 21), which are consistent with other published results [23], [31]. The number of Mi1 hair cells decreased as the ethanol concentration increased (Fig. S1A; n = 10-28). Similar to the O2 neuromast, there were significantly fewer hair cells (p<0.01) in ethanol-treated animals when compared to untreated controls at concentrations greater than 1% (Fig. S1A).

### Ethanol disrupts the development of functional lateral line hair cells

The voltage-sensitive dye FM 1-43FX passes through the mechanotransduction channel to label functional sensory hair cells in the lateral line of the zebrafish [26], [27], [37]. Untreated zebrafish (Fig. 2B) and zebrafish treated with 0.75% or 1% ethanol (Fig. 2C) had FM 1-43FX-labeled hair cells in neuromasts found around the head and along the length of the body. Higher concentrations of ethanol resulted in fish that were not labeled nearly as prominently. Fish from the 1.25% ethanol treatment had much less labeling in neuromasts found along the head and along the body and tail (data not shown). Fewer neuromasts were labeled in the head region of fish treated with 1.5% ethanol (Fig. 2D). The larvae raised in 1.75% ethanol had little to no labeling in the head region and along the tail, but their nasal epithelium were labeled with FM 1-43FX indicating that they were alive at the time of labeling [38]. The percentage of FM1-43 labeled cells that were co-labeled with GFP was calculated. The percentage of functional hair cells was significantly less than controls (p<0.01) in larvae at the highest concentrations (1.5% and 1.75%) of ethanol used (Fig. 2F). In the Mi1 neuromast, over 90% of the hair cells were labeled with FM-143FX in the untreated controls, 0.75%, 1%, and 1.25% ethanol conditions (n = 8-10 per condition). Similar to the O2 neuromast, there were significantly fewer functioning hair cells in the Mi1 neuromast at the highest two concentrations (Fig. S1B; p<0.01; n = 11-14).

### Methanol does not affect the development of lateral line hair cells

To determine whether the effects were ethanol specific [39], Brn3c-GFP zebrafish embryos were treated with 1% or 1.5% methanol from 2 to 5 dpf, stained with FM 1-43FX, fixed, and imaged. Methanol-treated larvae were indistinguishable from untreated controls; they swam normally and did not exhibit any observable dysmorphology (data not shown). There were no differences in the numbers of hair cells found in both the O2 and Mi1 neuromasts between untreated controls and larvae raised in 1% methanol (data not shown) or 1.5% methanol (Fig. 2E, Fig. S1A, p = 0.95, n = 8-12 per treatment group). Almost all lateral line hair cells in the methanol-treated embryos were labeled with FM 1-43 (greater than 98.5%) indicating that these hair cells had functioning mechanotransduction channels (Fig. 2F, Fig. S1B).

### Ethanol reduces the number of sensory hair cells by affecting both proliferation and cell death

**Figure 3:**
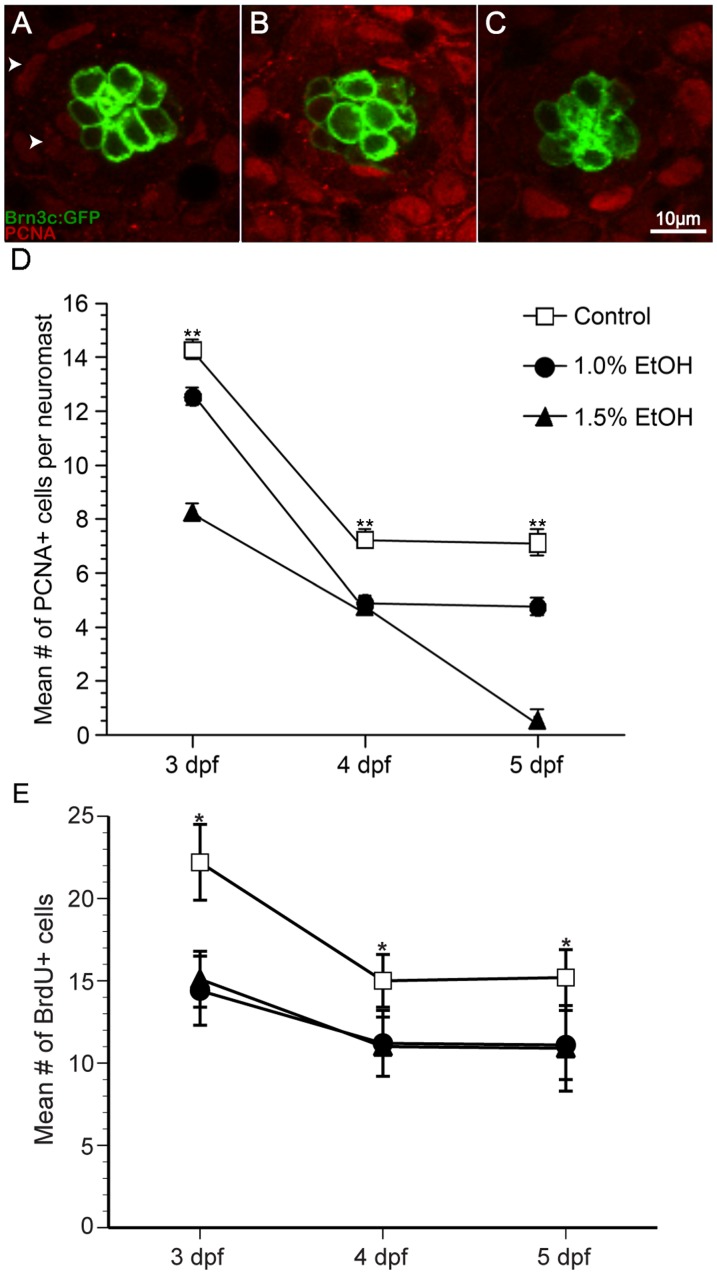
Ethanol exposure significantly reduced the number of proliferating cells compared to untreated controls in O2 neuromasts of Brn3c GFP-larvae. (A) Under untreated control conditions, proliferating cell nuclear antigen (PCNA)-labeled cells (arrowhead) are primarily non-sensory supporting cells and mantle cells in the neuromast (area demarked by white dotted line). Fewer PCNA-labeled cells result from ethanol treatment at (B) 1% and (C) 1.5% by volume. (D) The mean number of PCNA-labeled cells decreases during maturation and according dose of ethanol. (E) In a separate experiment, bromodeoxyuridine was added to the embryo medium during the last 24 hours of treatment, fixed, processed for BrdU immunohistochemistry, and BrdU-labeled cells were counted. Significantly fewer BrdU-labeled cells were observed in ethanol-treated animals when compared to untreated controls. Results are the mean values ± SD. n = 10-21 neuromasts for each treatment group. **p<0.01.

Since there was a reduction in the number of neuromast sensory hair cells, we hypothesized that the ethanol treatment caused a reduction in cell proliferation, an increase in the levels of cell death, or a combination of both. Supporting cells in lateral line neuromasts surround each sensory hair cell and are designated as either internal or peripheral (mantle cells) depending upon the location within the neuromast (Fig. 3A) [40]. The supporting cells serve as a source of new cells within the neuromast [40].

Ethanol-treated zebrafish were fixed at 3, 4, or 5 dpf and processed for proliferating cell nuclear antigen (PCNA) immunohistochemistry. PCNA labels proliferating cells G1 through G2 phase of the cell cycle [29], [41]. PCNA-labeled cells were observed in the periphery of the neuromast, where most cells are added (Fig. 3A). In control larvae, there was a significant decrease in the number of PCNA-labeled cells between 3 and 4 dpf (p<0.01; Fig. 3D) but little difference between 4 and 5 dpf in the O2 neuromast (Fig. 3C). Ethanol-treated larvae also showed a significant reduction in the number of PCNA-labeled cells when compared to untreated controls (p<0.01; Fig. 3B-D). In the 1.5% ethanol-treated animals, there was a continuous decrease in the number of PCNA-labeled cells (Fig. 3C, D). Moreover, there was a significant difference in the number of PCNA-labeled cells in the 1% and 1.5% ethanol-treated animals at each day (p<0.01; Fig. 3D).

Similar changes in PCNA labeling were observed in the Mi1 neuromast. There was a decrease in the number of PCNA-labeled cells in control larvae over time (Fig S2A). Significantly fewer PCNA-labeled cells were observed in both 1% ethanol (p<0.05) and 1.5% (p<0.01) when compared to untreated controls (Fig. S2A, n = 10-21).

To confirm the PCNA observations, the experiment was repeated but the S-phase maker bromodeoxyuridine (BrdU) was added to the media during the last 24 hours of the experiment. The larvae were fixed at 3, 4, or 5 dpf and processed for BrdU immunohistochemistry. The data obtained from the BrdU experiment were similar to the PCNA data in the O2 neuromast (Fig. 3E). Fewer BrdU-labeled cells were observed in untreated controls as the animals matured. There was a significant reduction in BrdU-labeled cells in ethanol-treated animals when compared to untreated controls in the O2 neuromast (p<0.05; Fig. 3E).

Similar changes in BrdU labeling were also observed in the Mi1 neuromast. There was a decrease in the number of BrdU-labeled cells in control larvae over time (Fig. S2B). Fewer BrdU-labeled cells were observed using 1% ethanol while 1.5% ethanol reduced the number of BrdU-labeled cells (Fig. S2B, n = 10-15).

**Figure 4:**
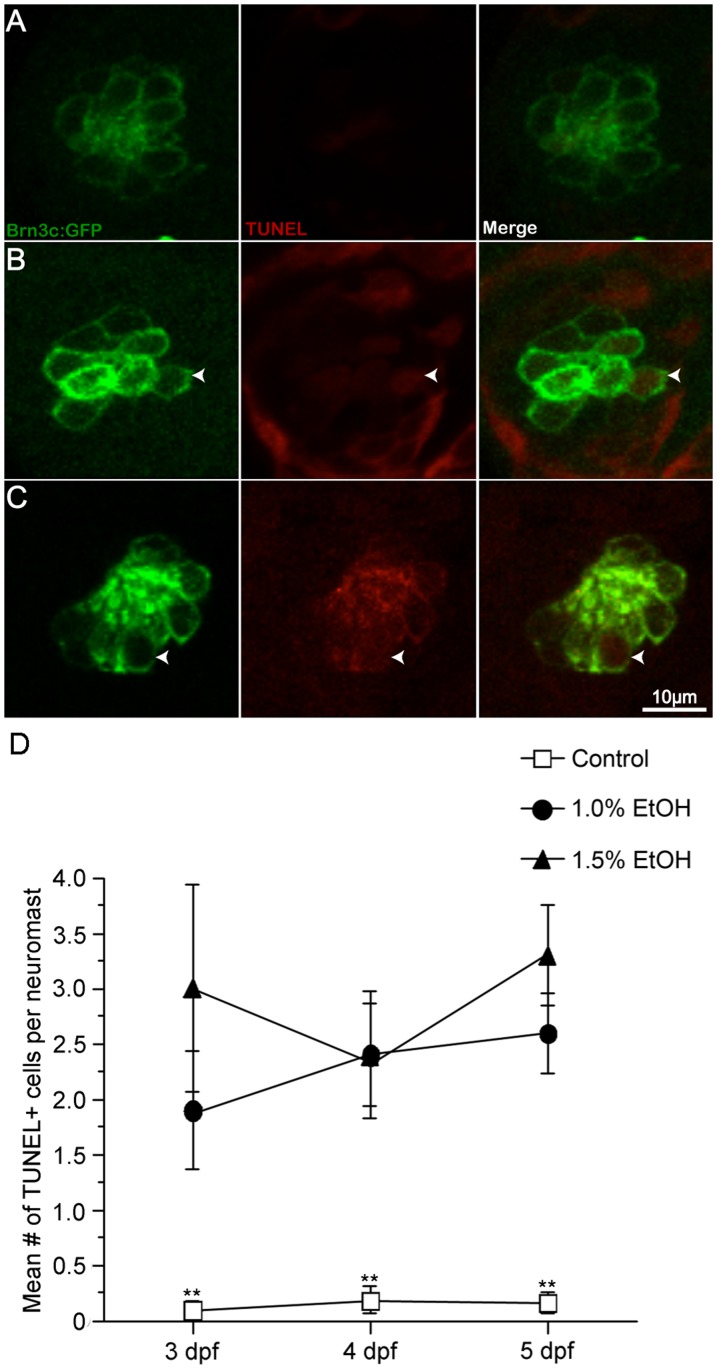
Ethanol exposure significantly increased the number of TUNEL-labeled cells in the O2 neuromast. Apoptotic activity in O2 neuromasts was measured by TUNEL labeling of untreated and ethanol-treated Brn3c-GFP larvae from 2-5 dpf. (A, D) Untreated controls showed few TUNEL-labeled cells but (B, D) 1% ethanol-treated and (C, D) 1.5% ethanol-treated larvae showed a significant increase in the number of TUNEL-labeled cells (arrowhead) versus control animals. While there were more TUNEL-labeled cells in ethanol-treated animals when compared to untreated controls, there was no statistical difference between ethanol concentrations. Results are the mean values ± SD. n = 9-12 neuromasts for each treatment group. *p<0.05 and **p<0.01 when compared to untreated controls.

In order to assess the relative levels of cell death, ethanol-treated fish from 3, 4, and 5 dpf were fixed and processed for TUNEL-labeling. Very few TUNEL-positive cells were observed at any time point in either the O2 (Fig. 4A). There was a significant increase in the number of TUNEL-positive cells at 3, 4, and 5 dpf in the ethanol-treated larvae when compared to untreated controls (p<0.01). There was, however, no statistical difference in the number of TUNEL-positive cells when comparing the two concentrations of ethanol (p>0.05; Fig. 4C).

Similar observations were observed in the Mi1 neuromast at 3, 4, and 5 dpf. There were 0-0.2 TUNEL-positive cells in untreated controls (Fig. S2C, n = 10-14). There were significantly more TUNEL-positive cells between 1.5% and untreated controls (p<0.01) at all three time points but only between 4 and 5 dpf in the 1% ethanol-treated animals (Fig. S2C, n = 9-14).

Since the lateral line hair cells were labeled with GFP, we were able to determine if the hair cells or some other cell type in the neuromast (presumably supporting cells) were TUNEL-positive. All of the TUNEL-positive cells were double labeled in control animals indicating that the few dying cells in the neuromast were hair cells confirming that some natural turnover occurs in neuromasts [42]. In ethanol-treated animals, most of the TUNEL-positive cells were double labeled at 3 dpf and 4 dpf (80–95% double labeled) in both neuromasts. Interestingly, a substantial number of single-labeled TUNEL-positive cells (30–40%) were found at both ethanol concentrations at 5 dpf indicating that ethanol may have toxic effects on cells other than hair cells in the O2 and Mi1 neuromasts.

### Lateral line hair cell populations do not recover following ethanol treatment

Since zebrafish can rapidly regenerate hair cells following certain ototoxic insults within 24–48 hours after insult [31], we assessed whether hair cell numbers returned to normal after ethanol treatment was stopped. Larvae were treated with ethanol from 2-5 dpf and then raised until 10 dpf in normal EM. The morphology of the 1% ethanol-treated fish did not change much after the cessation of the ethanol treatment but a percentage of the fish raised in the 1.25% ethanol began to resemble the 1.5% ethanol larvae at 5 dpf (see Fig. 1C for reference). There was a high attrition rate of embryos raised in 1.25% and 1.5% ethanol after 7 dpf (over 70% per condition) and all of the 1.5% ethanol-treated fish were dead by 8 dpf. The number of hair cells in the O2 and Mi1 neuromasts at 10 dpf was not significantly different than those found in ethanol-treated fish at 5 dpf (p>0.1). For instance, at 10 dpf, there were 12.4±0.9 hair cells in the O2 neuromast of untreated larvae (n = 12) but only 9.4±1.6 hair cells in embryos treated with 1% ethanol (n = 11) and 8.3±1 hair cells in embryos treated with 1.25% ethanol (n = 10) implying that the numbers of hair cells do not return to normal levels (Table 1). Similar hair cell counts were observed in the Mi1 neuromast (Table 1).

**Table 1:** Hair cell numbers do not return to normal levels following cessation of ethanol treatment.

	O2 neuromast		Mi1 neuromast	
	5 dpf	10 dpf	5 dpf	10 dpf
Control	13.1±2.3	12.4±0.9	12.8±1.8	11.1±0.92
1.00% EtOH	10.2±2.0	9.4±1.6	10.6±2.0	9.1±1.0
1.50% EtOH	8.9±2.1	8.3±1.0	9±2.3	8.2±1.1

Zebrafish were treated with ethanol from 2 days post-fertilization (dpf) to 5 dpf. Some animals were fixed and hair cell counts from the O2 and Mi1 neuromasts were obtained. Other embryos were raised in embryonic medium from 5 dpf to 10 dpf, fixed, and then hair cell counts were obtained. There were no significant differences in the number of hair cells within a specific condition between 5 dpf and 10 dpf larvae (p>0.1). Results are mean ± SD (n = 10-15 per condition).

## Discussion

Larval zebrafish treated with ethanol developed morphological abnormalities similar to those exhibited by children with FAS. Additionally, ethanol treatment during neuromast development resulted in a decrease in hair cell quantity and a decrease in the amount of functional hair cells. Methanol treatment did not affect the development of hair cells. Proliferation within a neuromast, as measured by PCNA and BrdU immunohistochemistry, also decreased in response to ethanol treatment. Finally, cell death within the neuromast, as assayed by TUNEL labeling, increased.

### Zebrafish as a model to study FAS

The ear, like the rest of the zebrafish develops rapidly; the ear is functional as early as four to five dpf [43]. The most notable advantage for using zebrafish as a model for hearing and auditory function is the fact that fish, like other aquatic animals, possess another system called the lateral line, which contains both hair cells and non-sensory supporting cells in neuromasts that reside along the head and side of the zebrafish. These hair cells are very similar in structure and function to inner ear sensory hair cells and develop along a similar time frame [32], [33]. Ethanol may be used to treat zebrafish embryos because ethanol passes unimpeded through the protective chorion surrounding the embryo during development [5]. Other studies have showed that zebrafish can successfully be utilized as a model system for FAS to study learning and memory, cell death in the CNS, skeletal dysmorphogenesis, and abnormalities in muscle development [1]. Moreover, in both humans and zebrafish, ethanol exposure during development causes physical abnormalities such as higher mortality, microphthalmia or small eyes, body distortions such as an enlarged body cavity, smaller body length, and changes in heart rate [5], [16].

Zebrafish, when treated with alcohol during development, exhibit many of the symptoms that occur in children with FAS. A recent study reported embryonic ethanol concentrations at 3.4 mM (0.015g/dl) following a 48-hour incubation in 251 mM ethanol (1.5%) [20] while another study measured a much higher concentration, 71 mM following 48-hour incubation in 200 mM ethanol [44]. In pregnant mammals, fetal alcohol concentrations matched maternal levels and only differed in time of onset [45]. In humans, an equivalent tissue concentration can be reached in a 130lb woman with just 3 standard alcoholic drinks but the mother would need to maintain this level by drinking regularly and substantially.

Interestingly, the ethanol levels necessary to recapitulate the FAS phenotype in zebrafish are an order of magnitude higher than the blood alcohol levels considered lethal in humans. Cold-blooded vertebrate embryos absorb ethanol along with oxygen and some nutrients through their skin hence, the levels of alcohol that reach the embryo’s blood stream may be within the levels to which mammalian embryos are exposed to during their development. The amount of ethanol that is toxic to the zebrafish is an order of magnitude lower after animals have developed gills (Christopher Lawrence, personal communication)[16].

Ethanol, however, causes developmental impairments in the zebrafish inner ear and lateral line [20]. In a recent study Zamora and Lu (2013) showed that ethanol exposure during early development reduced the size of the otoliths, the size of the neuromast as well as hair cells per neuromast, however, ethanol treatment began at 2 hours post-fertilization when most neuromasts were not formed [20]. Additionally, they did not investigate a mechanism for reduction in hair cells per neuromast or whether ethanol decreased the number of functional hair cells [20]. The data presented in the current study demonstrate that ethanol treatment results in animals with fewer hair cells per neuromast at 5 dpf, when these neuromasts are fully formed. Additionally, ethanol treatment results in decreased proliferation and greater cell death in these neuromasts.

Because FM 1-43 is a sensory cell loading dye, only functional hair cells uptake the dye and fluoresce [26], [27], [37]. We observed that the higher the concentration of ethanol treatment produced fewer functional hair cells in the developing fish. One possibility is that ethanol may delay the maturation of functional hair cells. Using different vital dyes, Santos and colleagues demonstrated that lateral line hair cells mature over a period of days [33]. Since we demonstrate that the number of cells do not change after the cessation of ethanol treatment, this reduces the likelihood of this as a reason.

Methanol, on the other hand, had no effect on the developing larvae as the animals resembled untreated controls and had no distinct differences in hair cell development, morphology or functionality. These observations are in line with previous research that observed no differences in gross morphology when animals were treated with methanol concentrations as high as 2% [46]. Also, methanol does not affect the morphology or function of photoreceptors in zebrafish [16]. Therefore, it appears that zebrafish are not a good model to study methanol toxicity that is observed in humans as it appears that methanol is metabolized differently between species.

### Ethanol affects proliferation levels and kills sensory hair cells

Sensory hair cells arise from non-sensory supporting cells in the lateral line [31]. In this study we show that alcohol could reduce supporting cell proliferation, which would reduce the total number of hair cells. Exposure to ethanol disrupts normal levels of proliferation of different neural precursors in the CNS. This disruption appears to be dose-dependent and varies between cell types and can result in increased [47], or more commonly, decreased proliferation [47], [48]. In both cases ethanol appears to alter cell cycle kinetics as well as change the proportion of cells that are actively cycling. Ethanol exposure during zebrafish retinal neurogenesis often results in reduced eye size and cyclopia at higher doses [16], [18], [49]. Additionally, these studies observed delayed differentiation in many retinal cell types including photoreceptors. Interestingly two studies saw either no difference or slight increases in proliferation in the ethanol-treated fish [18], [49]. Our data suggest yet another tissue-specific effect of ethanol on proliferation. We observed an overall decrease in cell proliferation within each neuromast as the animal matured in the control condition. In contrast, the ethanol-treated animals had fewer proliferating cells per neuromast at 3, 4, and 5 dpf, but still showed a similar decrease as the animal matured.

In addition to disrupting proliferation, ethanol administration has also been shown to increase apoptosis in the CNS of rats [50], mice [51], chicks [52], and zebrafish [1]. In the developing zebrafish retina only modest increases in cell death have been observed, when exposed to ethanol [18], [49]. The data presented in the current study shows significant increases in TUNEL-labeled cells in the neuromasts of zebrafish treated with ethanol. The combination of decreased proliferation of supporting cells and increased hair cell death are presumably the cause for significant reduction in the amount of mature hair cells per neuromast in the ethanol treated animals.

### Retinoic acid as a potential mechanism regulating the decrease in the number of lateral line sensory hair cells

Ethanol, a substrate for alcohol dehydrogenase, may derive its teratogenicity by competitively inhibiting alcohol dehydrogenase catalyzed retinoic acid synthesis [53]. The vitamin A derivative retinoic acid plays a key role in anterior-posterior patterning as well as development of the hindbrain [54]. Depletion of retinoic acid during key developmental windows results in malformations of the face, neural crest, eyes, heart, and nervous system [54], [55]. Previous work has also shown that retinoic acid plays a key role in hair cell differentiation and sensory epithelial development [56], [57]. Application of exogenous retinoic acid to embryonic mouse cochlear cultures results in supernumerary hair cells [56]. Antagonism of the retinoic acid receptors or inhibition of retinoic acid synthesis in the developing cochlea results in a reduction of differentiated hair cells [57].

The rate limiting step of retinoic acid synthesis, the oxidation of retinol to retinal, is catalyzed by alcohol dehydrogenase. Alcohols, including ethanol, are also substrates for alcohol dehydrogenase and can therefore competitively inhibit synthesis of retinoic acid. High dose ethanol treatment of cultured mouse embryos has been shown to reduce detected retinoic acid levels [58]. We have presented in this current study a model for fetal alcohol syndrome induced hair cell loss. A potential mechanism for the reduction in hair cells is inhibition of retinoic acid synthesis via competition for alcohol dehydrogenase by retinol and ethanol. A similar mechanism was proposed for ethanol-induced microphthalmia, reduced eye size, in zebrafish however, recent findings suggest that alteration of retinoic acid signaling may not play a role in ethanol as a teratogen [19]. Other potential implicated mechanisms of ethanol-induced cell death include but are not limited to increased oxidative stress [59], epigenetic alterations [60], and increases in intracellular calcium [61]. Future studies investigating the mechanism of ethanol-induced hair cell death will have to take all of this previous work into consideration when investigating how ethanol treatment during development results in fewer hair cells per neuromast.

## Supporting Information

Figure S1 **Ethanol but not methanol treatments affect the number of functional sensory hair cells in the Mi1 neuromast.** Larval zebrafish were treated with ethanol, methanol, or embryo medium beginning 2 days post-fertilization (dpf) through 5 dpf. Larvae were briefly exposed to FM 1-43FX before fixed, mounted, and the Mi1 neuromast was imaged. (A) Significantly fewer hair cells were observed in the Mi1 neuromast of animals treated with 1.25%, 1.50%, and 1.75% ethanol when compared to untreated controls. (B) The percentage of GFP-labeled cells that were also co-stained for FM 1-43 decreased as the ethanol concentration increased but not in the methanol treatment group. There was a significant decrease in the number of double-labeled hair cells at the two highest concentrations of ethanol tested. Results are the mean values ± SD. n = 8-28 per condition. **p<0.01 when compared to untreated controls.
(TIFF)

Figure S2 **Ethanol exposure reduced the number of proliferating cells and increased the number of TUNEL-labeled cells in the Mi1 neuromast.** Larval zebrafish were treated with ethanol or embryo medium beginning 2 days post-fertilization (dpf) and larvae from each group were fixed at 3, 4, or 5 dpf. Images and cell counts were taken of the Mi1 neuromast. (A) Fewer PCNA-labeled cells were observed following treatment with 1.00% or 1.50% ethanol treatment when compared to controls for 3, 4 and 5 dpf animals. (B) The mean number of BrdU-labeled cells in larvae treated with either 1.00% or 1.50% ethanol decreased when compared to untreated controls at 3, 4 and 5 dpf. (C) There was an increase in the number of TUNEL-labeled cells in larvae treated with 1.00% ethanol at 4 and 5 dpf but there was a significant increase in the number of TUNEL-labeled cells at all three time points in larvae treated with 1.50% ethanol. Results are the mean values ± SD. n  = 9-21 per condition. *p<0.05; **p<0.01 when compared to untreated controls.
(TIFF)
