# Supplementary figures and images for: Ethanol Affects the Development of Sensory Hair Cells in Larval Zebrafish (Danio rerio)
Source: PLoS One. 2013 Dec 6;8(12):e83039. doi: 10.1371/journal.pone.0083039 (PMC3855788; doi:10.1371/journal.pone.0083039)

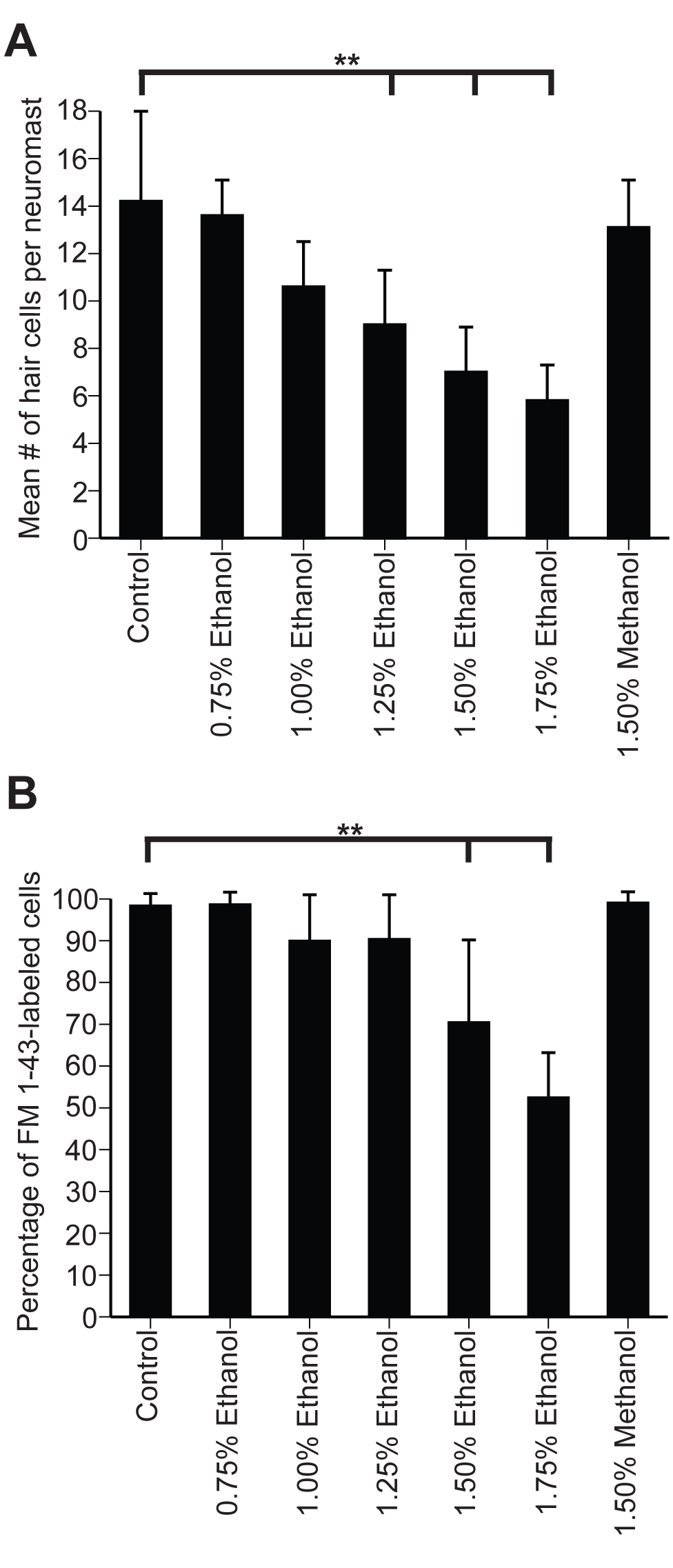

Supplement: Figure S1 — Ethanol but not methanol treatments affect the number of functional sensory hair cells in the Mi1 neuromast. Larval zebrafish were treated with ethanol, methanol, or embryo medium beginning 2 days post-fertilization (dpf) through 5 dpf. Larvae were briefly exposed to FM 1-43FX before fixed, mounted, and the Mi1 neuromast was imaged. (A) Significantly fewer hair cells were observed in the Mi1 neuromast of animals treated with 1.25%, 1.50%, and 1.75% ethanol when compared to untreated controls. (B) The percentage of GFP-labeled cells that were also co-stained for FM 1-43 decreased as the ethanol concentration increased but not in the methanol treatment group. There was a significant decrease in the number of double-labeled hair cells at the two highest concentrations of ethanol tested. Results are the mean values ± SD. n = 8-28 per condition. **p<0.01 when compared to untreated controls. (TIFF) [file pone.0083039.s001.tiff]

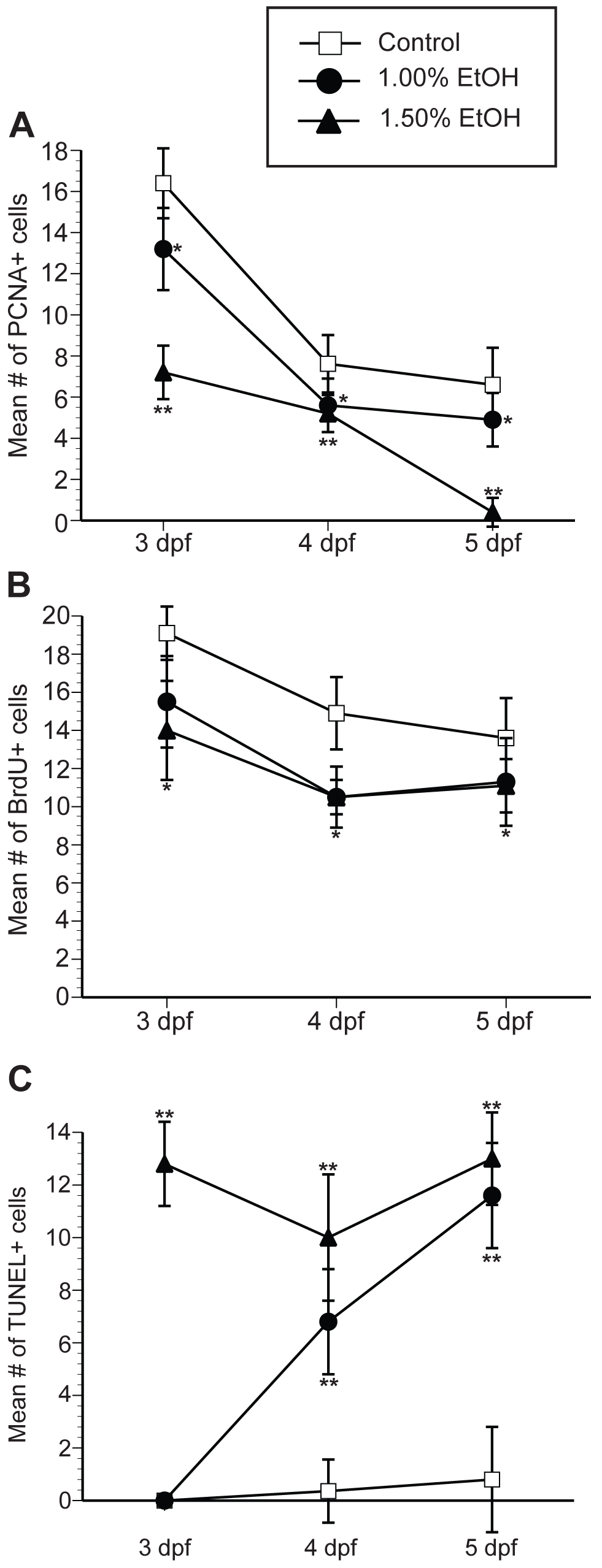

Supplement: Figure S2 — Ethanol exposure reduced the number of proliferating cells and increased the number of TUNEL-labeled cells in the Mi1 neuromast. Larval zebrafish were treated with ethanol or embryo medium beginning 2 days post-fertilization (dpf) and larvae from each group were fixed at 3, 4, or 5 dpf. Images and cell counts were taken of the Mi1 neuromast. (A) Fewer PCNA-labeled cells were observed following treatment with 1.00% or 1.50% ethanol treatment when compared to controls for 3, 4 and 5 dpf animals. (B) The mean number of BrdU-labeled cells in larvae treated with either 1.00% or 1.50% ethanol decreased when compared to untreated controls at 3, 4 and 5 dpf. (C) There was an increase in the number of TUNEL-labeled cells in larvae treated with 1.00% ethanol at 4 and 5 dpf but there was a significant increase in the number of TUNEL-labeled cells at all three time points in larvae treated with 1.50% ethanol. Results are the mean values ± SD. n = 9-21 per condition. *p<0.05; **p<0.01 when compared to untreated controls. (TIFF) [file pone.0083039.s002.tiff]
